# A Situational Mapping Overview of Training Programmes for Community-Based Rehabilitation Workers in Southern Africa: Strategies for Strengthening Accessible Rural Rehabilitation Practice

**DOI:** 10.3389/fpubh.2020.569279

**Published:** 2020-11-03

**Authors:** Lieketseng Ned, Ritika Tiwari, Lucia Hess-April, Theresa Lorenzo, Usuf Chikte

**Affiliations:** ^1^Centre for Rehabilitation Studies, Department of Global Health, Stellenbosch University, Cape Town, South Africa; ^2^Division of Health Systems and Public Health, Department of Global Health, Stellenbosch University, Cape Town, South Africa; ^3^Department of Occupational Therapy, University of the Western Cape, Cape Town, South Africa; ^4^Division of Disability Studies and Inclusive Practices Africa, Department of Health and Rehabilitation, University of Cape Town, Cape Town, South Africa; ^5^Division of Health Systems and Public Health, Department of Global Health, Stellenbosch University, Cape Town, South Africa

**Keywords:** community-based rehabilitation, community rehabilitation worker, disability, training, rurality

## Abstract

In 2018, the United Nations global report showed that people with disabilities, who make up 15% of the worlds' population, have poorer health and rehabilitation access (SDG 3). Without improving the needed person-centered health and rehabilitation services at household level, SDG 3 cannot be achieved. This includes addressing human resource shortages through training multi-skilled community based rehabilitation workers (CRWs) to build rural workforce capacity and enhance the lives of people with disabilities, particularly in LMICs where the need is higher but resources are lower. However, to date, there is no documentation and analysis of existing training and its scope for this workforce in LMICs. A situational mapping overview was undertaken to review the current status of rural rehabilitation training programs offered in Southern Africa for CRWs. CRWs are rehabilitation personnel, based in the home/community, who are not professionals (without a bachelor qualification) but render non-institutional rehabilitation and inclusive development in communities, under the supervision of rehabilitation practitioners. Information on these programs was obtained using a two-step process. Firstly, a descriptive list of university courses for rehabilitation workers offered in the Southern African countries was collected via an internet and literature search. Secondly, detailed information about the disability and rural rehabilitation courses was collected from the respective institutions and their designated websites. There are six training courses targeted at CRWs or disability practitioners with a disability focus being offered at universities in Southern Africa, five of these in South Africa and one in Zimbabwe. Additionally, four training courses are offered as online/open resources by global organizations and are self-directed with no accreditation. While other key competencies feature, none of these programmes' learning outcomes make direct reference to the rural practice context and its complexities in relation to disability and poverty. The situational mapping overview shows limited training targeted at CRWs in Southern Africa, to effectively facilitate rural rehabilitation, poverty reduction and social inclusion. There is a need for an articulated community-orientated rural training to respond to the unmet needs. This may require a different set of competencies and assessment standards for trainees as well as additional competencies for their supervisors and mentors.

## Introduction

In the United Nations Convention on the Rights of Persons with Disabilities (UNCRPD), disability is defined as “those who have long-term physical, mental, intellectual or sensory impairments which in interaction with various barriers may hinder their full and effective participation in society on an equal basis with others” (1). The World Report on Disability estimates that 15% of the world's population has some form of disability, and of those, 80% live in low-income countries with little or no access to basic health and social services (2). While a higher proportion of people with disabilities live in relative poverty in both high and low-middle-income countries, rural contexts, particularly, tend to have higher prevalence of disability because of the intersection between disability, poverty and rurality which heightens the barriers to health, rehabilitation, education and work (3–9). Recent data from nine countries in Southern Africa suggests that an average of 64% of people with disabilities who need rehabilitation are not able to access these services (10)–this is an ongoing problem in rehabilitation practice and the situation may be dire in rural contexts.

Adequate numbers of available trained workers who deliver rehabilitation services is an important proxy for ensuring the realization of rehabilitation goals within the context of human rights especially in low resource settings. Consistent with literature, the demand and need for rehabilitation is much higher than what can be provided by available services in low- and lower-middle-income countries (LMICs) (11). This is compounded by a low density of well-trained rehabilitation practitioners required for the delivery of adequate services (12–14). The situation remains worse in rural contexts (15, 16). Consequently, human resource analysis shows higher disability prevalence and higher service demands in contexts with shortages of human resources (11). While many human resource national plans and reviews tend to leave out rehabilitation (2), studies in countries like in South Africa (17–19), in Malawi (20) and in Kenya, Tanzania and Uganda (9, 21) indicate this field and its workforce constraints in less resourced settings (22).

Given that shortages of appropriately trained and deployed human resources is one of the bottlenecks for expanding access to rehabilitation services (14), one way of decentralizing and expanding service delivery is a deliberate focus on human resources at home and community levels in order to increase the supply of and access to rehabilitation closer to where people live (14). With the specialized skill and experience of working at household and community level, CRWs are the most cost-effective resource who could play a role in strengthening access and services, particularly in Africa where such workers are already in rural communities and, with training, can provide independent rehabilitation services, over recruiting workers from outside the community (23). However, a recent systematic review on the effectiveness of alternative cadres in community based rehabilitation (CBR) shows that, there is a need for more research on the training, development of these workers (22).

In the context of community based rehabilitation practice, mid-level workers are generally known as community-based rehabilitation workers(CRWs) (24) or community rehabilitation facilitators (CRFs) (12) or community disability workers (CDWs) (25). For the purpose of this paper, the term community-based rehabilitation workers (CRWs) will be used as a generic term to refer to these workers. CRWs are rehabilitation personnel, based in the home and/or community levels, who are not professionals (without a bachelor qualification) but render person-centered and community-based rehabilitation and inclusive development at home under the supervision of rehabilitation practitioners (with a bachelor qualification based in district hospitals), thus compensating professional human resource constraints and improving access to rehabilitation and health services particularly in rural contexts (24, 26). They work across the community non-hospital based health, education, labor, social and development sectors as guided by community based rehabilitation (CBR) Guidelines (27) to ensure effective implementation of community based inclusive development (CBID) (20, 21). CRWs, as key drivers of CBR, were introduced into community based rehabilitation as part of an interdisciplinary rehabilitation team with their role including following-up of clients seen at primary health care (PHC) level services (28, 29). Being primarily community based, CRWs engage and support people with disabilities, their families and communities in a range of rehabilitative, education and advocacy activities, frequently aimed at maximizing inclusion, social integration and participation (30). In addition, their work also puts emphasis on disability on the facilitation of empowerment of people with disabilities and their families for social and economic inclusion of people with disabilities (27).

Against this background, much is already known about the impact of CRWs, inclusive of rural settings. For instance, Chappell and Johannsmeier (12) showed that these workers transcended the result of individual medical rehabilitation in South Africa to include aspects of community development and equalization of opportunities which involved poverty reduction and social inclusion. Another study in three rural communities in Botswana, South Africa and Malawi found that these workers to be bridging the gap between people with disabilities, their families and services at district level (31). Working at community level, they are also able to assist authorities to identity, screen, build trusting relationships and support people with disabilities and their families (32) thus facilitating the restoration of dignity and a sense of belonging (12). Furthermore, a national study (24, 33) in South Africa demonstrated the role played by CRWs in reducing inequalities in access to health and social services through coordinated actions toward mobilizing resources across different government departments. A common finding on these studies is that, for communities where CRWs were present, people with disabilities had better access to education, healthcare and other social services (24, 33). This ensures that people with disabilities are not left behind. Given the resourcefulness of these workers, what training is available to build the skills of this workforce and strengthen the already under resourced rehabilitation practice? What learning outcomes are targeted by such available training? And can the training improve rural practice?

### Training Needs of Community-Based Rehabilitation Workers

The shortage of rehabilitation workers to address rehabilitation needs at a community level underlines the need for the development of education and training programs for community-based rehabilitation workers. Training programmes need to be regularly reviewed to ensure their continued relevance. Given that gaps in service-delivery, as perceived by people with disabilities, include poor identification of needs, not having basic needs met and inadequate community interventions (12), deficiencies in services such as health care, rehabilitation, social support and assistance combined with financial constraints and inadequate and inaccessible transport (8, 34), it would be crucial that training of CRWs prepares them to respond accordingly. For example, as Chappell and Johannsmeier (12) further emphasized, training needs to address development and barriers at the community level rather than solely focusing on impairment needs, more clinically—oriented interventions at home. Supporting this, a recent scoping review on the training needs for CBR workers not only highlights the need for training in clinical, management, and cultural competence skills but also the need for competencies such as empowerment of people with disabilities and community development practice skills to be addressed in training programmes (35). As such, CBR training assumes significance as gaps highlighted toward developing skills for a critical practice of rehabilitation include tenets of disability inclusion, human rights and social justice. Additionally, such training needs to equally address the need for ongoing professional development of CRWs. For example, adopting flexible and blended training approaches could facilitate the needed ongoing development (35) because it moves away from training that is not solely based in urban centers.

The development of these workers also requires that they attain specific relevant competencies. One study which explored the competencies that could strengthen the training of CRWs for disability-inclusive development in rural areas of South Africa, Botswana and Malawi identified a unique set of competencies (36). These competencies included; integrated management of impairment needs with a focus on functionality, advocacy, negotiation, networking, empowerment and capacity building skills (36). The study reiterated that it is imperative for training programmes to build skills in inter-sectoral and inter-disciplinary collaborative practice with specific reference to the complexities that rural contexts pose to the work of CRWs. In a different context, in Australia, the Rural and Remote Allied Health Competencies—Senior Professional (RRAHC-SP) framework suggests 88 competencies under 33 sub-domain and eight overarching domain areas. The eight domains include: service delivery, equity and diversity, professional skills, ethical practice, development and support, quality and safety, clinical management and clinical skills (37). While rural contexts are not homogenous, these competencies relate to the ones identified in the Southern Africa study and are responsive to the needs identified above.

However, there is a dearth of information on what current training of CRWs is available in the Southern region and secondly, how the training addresses needs and competencies required for under-resourced rural settings. Additionally, there remains an unanswered question on whether the training of CRWs should focus on developing generalist or specialist competencies for practice in rural contexts. Therefore, the aim of this study was to conduct a situational mapping overview of the current status of training offered for community based rehabilitation workers in Southern Africa, with a particular focus on advocating for training that capacitates CRWs' understanding of CBR and skills to effectively carry put their roles in response to the needs of people with disabilities in rural contexts. The objectives were to:

- Explore the programmes' admission requirements, mode of learning, course description, length and learning outcomes
- Propose strategies for development of community based rural rehab workforce and practice through training.

## Materials and Methods

This was a situational mapping overview (38) study aimed at mapping out existing training programmes for community-based rehabilitation workers. Such mapping categorizes literature, examines a situation in order to understand it and helps to identify gaps on a particular topic from which to conduct further research or reviews (38). The data regarding the existing CBR and disability inclusive development courses were obtained using a two-step process and the information was entered into a matrix.

As the first step, we created a list of courses offered in the Southern African countries i.e., Angola, Botswana, Lesotho, Malawi, Mozambique, Namibia, South Africa, Swaziland, Zambia, and Zimbabwe. We conducted an internet search using the Google search engine. Published literature was utilized to direct us to institutions offering disability and rural rehabilitation programmes and websites of various universities and institutions searched. Only certificate and diploma courses ranging in duration from short-term (3 months) to longer term (2 years) were included in the analysis. Keywords used for the search included “Community based rehabilitation,” “disability inclusive development,” “Community based rehabilitation workers,” “Southern Africa,” “Community-based rehabilitation OR training programme,” “Community-based rehabilitation OR training course,” “Community-based rehabilitation workers OR training OR education,” Community-based rehabilitation OR disability inclusive development course”; Community-based rehabilitation OR disability inclusive development training course” These specific phrases were searched independently and combined using connecting words.

In the second step, detailed information about the disability and rural rehabilitation programmes was collected from the respective institutions and from the designated websites of these institutions. The institutes with limited information were contacted through e-mails to get more information regarding the available programs, eligibility criteria, duration, nature of the program etc. Websites from specific NGOs with information about CBR and disability inclusive development training courses, documents of training manuals/tool-kits, and reports about training were also searched. NGO websites that provided freely accessible on-line manuals/toolkits, which tend to be more generic, focusing on the CBR guidelines or principles of disability and inclusion, and not tied to a specific country, e.g., Light for the World's CBR manual and CBM's Disability and Inclusive Development toolkit were also reviewed.

The collected data or information was incorporated into a matrix and the findings were triangulated wherever possible. Learning outcomes were accessed through each programmes' website, and analyzed through a deductive thematic analysis process. This implies that pre-defined codes (as shown in **Table 3**) were drawn from literature on CBR worker competencies and training needs. Each of the programmes' learning outcomes were assessed against these pre-defined codes. All data was available in the English language. All programmes that were not eligible to this level of workers and training that was toward a professional degree were excluded.

Ethics approval was obtained from the Stellenbosch University Health Research Ethics Committee (HREC Reference No: X19/03/007).

## Results

The paper reports the findings of the ten training courses being offered in CBR and disability inclusive development in Southern Africa in the year 2019–2020. Table 1 displays details of programmes, entry requirements, course information, and learning outcomes. Table 2 displays open source training resources available.

**Table 1:** Details of formal training currently available.

**No**	**Programme**	**Admission requirements**	**Course info**	**Learning outcomes**
1.	Post-graduate Diploma in Disability and Rehabilitation Center for rehabilitation studies, University of Stellenbosch, South Africa	A bachelor's degree or equivalent, qualification at National Qualifications Framework level 7, or Recognition of prior learning (RPL) where in some or other manner attained, in a particular field of study, a standard of competence.	Focuses on strengthening and deepening knowledge and theoretical understanding of disability and rehabilitation, with the aim of promoting the development of current thinking, response and practice in disability and rehabilitation studies. Comprehensive rehabilitation education and training programmes, research and community interaction opportunities for all health sciences and rehabilitation-related professionals at all levels of health services and in the community. Duration: 1 year	•Demonstrate responsible participation in the promotion of the quality of life and full inclusion of all persons with disabilities in the local, South African and global community. •Demonstrate sensitivity to, and strive for a deep understanding of cultural, religious, social and ethnic diversity and its impact on the disabled person. •Identify and find solutions to disability and rehabilitation-related problems through thinking within an outcomes-based approach •Work effectively with persons with disabilities, disabled persons organizations and other community groups; •Demonstrate familiarity with the legislation, policy documents and research literature in the field of disability and rehabilitation, and critically relate relevant literature to individual scope of practice; •Identify and define complex problems within the disability and rehabilitation scope of practice, and apply appropriate knowledge and skills to solve them; •Identify contradictions, challenge orthodox theory and practices, and suggest new approaches in the field of health, disability and rehabilitation; •Demonstrate comprehensive knowledge of the programme delivery principles, concepts and models in the field of disability management and rehabilitation, as well as the various contexts at primary, secondary and tertiary level in which these apply; and •Demonstrate mastery of advanced theory and its application to the specialized field of disability and rehabilitation.
2	Post-graduate Diploma in Disability Studies, Disability Studies division, Department of Health and Rehabilitation Sciences. UCT. South Africa	An undergraduate degree or equivalent in any discipline. Applicants who do not have an undergraduate degree may apply for admission on the basis of Recognition of Prior Learning (RPL) e.g., experience in the field of disability and development;	•The PG Diploma in Disability Studies programme aims to increase awareness and informed participation in disability issues at a teaching, research and community-based programme level. •Duration: 1 year	•Understand disability as an issue of diversity with deep psychological roots that results in social injustice because of power and privilege that favors the non-disabled norm •Be able to critically engage with research in the light of the transformative aims of the disability practitioner •Be familiar with the discourse of the discipline of Disability Studies with conceptual understanding and the ability to communicate understanding, thinking and reasoning in academically rigorous ways •Be able to monitor the capacity of government and development agencies to implement strategies that lead to the equalization of opportunity and social justice for disabled people •Be able to understand theories of development and how disability can be mainstreamed within these processes
3	Higher Certificate in Disability Practice, Disability Studies division, Department of Health and Rehabilitation Sciences. UCT. South Africa	Matric certificate or National Senior Certificate for Adults (NASCA) or HEQSF level 4 equivalent qualification; RPL	The programme creates foundational skills for disability prevention and care. This qualification is to provide students with the basic knowledge, cognitive and conceptual tools and practical techniques for application in the field of disability inclusive development. Duration: 1 year	•Select and screen disabled clients for impairments and provide basic interventions to improve participation in the life areas of living, learning, working and socializing. •Implement health promotion actions, education and strategies. •Promote the rights of people with disabilities and implement strategies and actions to enable participation. •Describe basic information systems and implement communication systems in relation to care pathways of people with disabilities. •Screen, provide basic care, and implement follow up and referral systems, as they relate to the needs of people with disabilities.
4.	Attendance Certificate: Short Course: The basics of Disability, Center for rehabilitation studies, University of Stellenbosch, South Africa	Grade 12/Matric Certificate	Students are introduced to disability basics Duration 3 days	•How the different disability approaches impacts the nature and experience of disability •The difference between disability, impairments and health conditions •The relationship between poverty and disability •The role of health care in disability
5	Attendance Certificate: Short Course on disability rights in an African context, Center for Human Rights, University of Pretoria	Practitioners working with persons with disabilities, human rights activists. Proficient in English.	The course promotes disability rights in Africa by raising awareness about the United Nations Convention on the Rights of Persons with Disabilities (CPRD) (2006) and the Protocol to the African Charter on Human and People's Rights on the Rights of Persons with Disabilities in Africa (2018). Duration: 1 week	•Understand the development of disability as a global human rights issue •Understand and apply provisions of the CRPD •Apply the CRPD to selected areas •Understand the development of disability as a human rights issue as the African regional and sub-regional levels •Understand and apply the intersection between the human rights and cultural aspects of disability in an African context •Understand and apply disability from a comparative human rights law perspective in other regions, including the European and Latin American regions •Understand and apply the regulation of disability from a comparative law perspective in selected non-African countries •Understand and apply theoretical approaches to equality and non-discrimination in a disability context.
6	Diploma in Disability Studies Zimbabwe open university	A relevant first degree pass Or accreditation of prior learning	Introduction to disability and disability issues Duration: 2 years	•Sensory, physical, motor and intellectual disabilities •Inclusion advocacy and empowerment •Communication with and counseling of people with disabilities •Community Development •Assessment and Rehabilitation •Legal and ethical issues of disability

**Table 2:** Other informal open source online training and training resources available.

	**Organization**	**Resource**	**Description**	**Learning outcomes**
7	CBM (Christian Blind Mission)	CBM Disability Inclusive Development Toolkit	The toolkit is designed as a resource that can be used in a variety of ways: to support staff induction, team meetings, refresher days and training workshops. It can also be used as a tool for personal reflection, self-study and a training resource. Has in-house informal training including supervision. Targets community members living within walking distances of clinic (persons with disabilities, mothers of children with disabilities or a disabled family member).	•Promote and apply disability inclusion, in work place, at home, in the community
8	WHO	INCLUDE Online Learning community (CBR)	An online learning community for community-based rehabilitation (CBR) that aims to inform and support CBR managers and interested stakeholders around the world It is an online programme that guides the user through different information modules based on the CBR guidelines: health, education, livelihood, social and empowerment.	•Learn about Community-based Rehabilitation (CBR) as an inclusive development strategy to realize the rights of people with disabilities at the community level •Discover how other programmes are putting CBR's inclusive development strategy into action •Create your own action plan for inclusive development •Share experiences, thoughts and ideas with a community of other dedicated individuals working in CBR •Reflect on your own experiences and beliefs about inclusive development
9	Light for the World	CBR Training manual	Build on existing basic CBR skills. The training manual covers organizational skills, knowledge and attitudes needed when implementing CBR in accordance with the various components of the CBR guidelines.	•Understand the structure of the guidelines document. •Know how to incorporate the CBR guidelines within the local context of their individual projects. •Use the guidelines as a tool to develop and implement their CBR projects.
10	Online Global Health and Disability Course London School of Hygiene and Tropical Medicine	Anyone with a professional or personal interest in disability as it relates to health, rehabilitation, international development and humanitarian assistance.	The course aims to raise awareness of the importance of the health and well-being of people with disabilities in the context of global development. There is a particular focus on low and middle income countries–both in the content of the course and the target learners. Duration: Minimum 5 weeks (online)	•Describe the links between disability, health and well-being •Discuss challenges to health and well-being amongst people with disabilities •Develop an understanding of what disability is and its relevance to the global development agenda •Reflect on how different types of disabilities affect people's lives in different ways •Identify solutions to improve health and well-being amongst people with disabilities

### Course Descriptions

There are 6 formal training retrieved courses targeted at community-based rehabilitation workers or disability practitioners with a disability focus being, offered at universities in Southern Africa. Five of these are offered in South Africa and one in Zimbabwe. While these 6 formal courses require a graduate degree as an entry requirement, they are also open to people who only have relevant years of experience with no qualifications. This implies that those who have a high school level of education and some experience in the field of disability and rehabilitation are also eligible. In the absence of proof of such formal qualifications, course participants are selected based on the recognition of their prior learning (RPL) in their particular field or area of disability and rehabilitation practice.

Additionally, 4 training courses are offered as online/open resources by global organizations. These courses also target community-based rehabilitation workers, people with disabilities and people working for NGO's but do not provide a formal qualification or accreditation as they are less formal, have no eligibility and are self-directed. The less formal training focus more on improving people's practical skills.

### Mode of Learning

All the formalized training programmes are offered by institutions of higher learning which are public universities. All the currently existing programmes, offer their courses in blended learning mode. All the formal programmes happens at institutional level. Apart from the informal training by CBM, none of the other programmes take place at community level or in rural contexts. While the higher certificate offers practice learning at community levels of care, this remain limited to urban settings due to the situatedness of the programme. The informal online programmes are self-directed, implying that CRWs within rural contexts could access these programmes as part of continued professional development.

### Scope of the Programmes' Learning Outcomes

Competencies that were listed by most courses encompassed an understanding of the concept of disability inclusion and skills related to clinical care or rehabilitation, health promotion, and community participation. Knowledge on disability and diversity as well as disability legislation were also listed by the majority of programmes (See Table 3). Fewer programmes listed competencies such as knowledge related to advocacy, cultural awareness, human rights, social justice, disability information and management as well as disability research. Only one programme specified knowledge on the CBR guidelines, while other competencies that were listed by the least number of programmes included monitoring and evaluation, ethical practice and capacity building of people with disabilities. None of these programmes' learning outcomes make direct reference to the rural practice context and its complexities.

**Table 3:** Themes emanating from Programmes' outcomes.

**Codes**	**Programmes**
Advocacy	1, 6
Disability inclusion	1, 2, 4, 5, 6, 7, 8
Clinical skills	1, 2, 3, 6
Health promotion	1, 3, 4, 10
Disability and diversity	1, 4, 5
Community participation	1, 7, 8, 6
Cultural awareness	1, 5
Human rights	3, 5
Principles of programme delivery	1
Social justice	2, 7
Capacity building	6
Networking and referral	3
CBR guidelines	9
Disability information and management,	1, 3
Ethical practice	6
Legislation on disability	1, 5, 6
Monitoring and evaluation	2
Disability research	1, 2

## Discussion

Our intention with this situational mapping overview was to determine the current status of existing training for CRWs, with a particular focus in rural practice. At the core of decentralizing and expanding rehabilitation services is a deliberate focus on human resources at the community level (14). Additionally, development of a skilled rural workforce through training rural people in rural settings to deliver the services needed in the community is a tenet of rural training pathways (39). Training of CRWs is thus recommended and advocated for, as a cost-effective strategy for developing and scaling up rehabilitation workforce for rural practice (18, 23, 25). Against this background, a number of points can be drawn from our findings.

The first is the reiteration of the limited training opportunities for CRWs not only for initial training but also limited opportunities for upskilling. Given that there are limited numbers that could enter through recognition of prior learning per class and that rehabilitation needs remain unmet (10), it may mean that we are not producing enough workforce. It is worth noting that the existing formal programmes accept candidates who have a prior degree but also have an allowance for limited numbers of those who assume entry by recognition of prior learning. It can thus be concluded that the training is limited for Southern Africa. For instance, while five of the programmes are located in South Africa, they are not only accepting South African students but international students. Compounding this issue is the major challenge that Universities often do not recognize certificate programs, as career pathways for CWRs (36) hence they are few. This is because this level of training is usually placed at Technical Vocational Education and Training (TVET) and Universities of Technology levels which offer certificate programs. This policy decision could be the reason as to why universities have been slow in investing on this level of training. In South African specifically, the discipline-specific training of assistants or technicians were similarly terminated and no Higher Education institution (HEI) has taken up this training (18). A compounding factor is that most NGOs that used to train have discontinued training for CRWs in contexts like South Africa due to lack of support and funding constraints (28, 40). Another contributing factor is the way multidisciplinary workers have gained inadequate support from governments in Southern Africa) (41).

Provided that TVET are supported with relevant human resources for training posts and collaborations with the rehabilitation practitioners, they could be best suited to facilitate this community-based training as they are located in rural communities. Currently, universities such as Cape Peninsula University of Technology and Walter Sisulu University in South Africa (SA) already have health promotion qualifications. However, no capacity has been built in other service levels to better support and supervise these workers and for continuity. Additionally, human resources for training remain a challenge as these PHC platforms have fewer rehabilitation practitioners both for service delivery and to do the training of CRWs. Elsewhere, partnerships were noted as central to effective delivery of decentralized training for medicine students (42). The Western Cape province (South Africa), as an example, recognized rehabilitation care workers as an essential resource capable of strengthening PHC and CBR across service platforms (43) and this could be a replicated strategy in other countries. Integral to recognition of this essential resource is an establishment of functioning partnerships between health and rehabilitation sectors, Universities of Technology, TVET and communities to increase uptake of training and successfully implement such decentralized training for CRWs.

This strategy does not mean that the process will be seamless. A succeeding issue would be that of country capacity to create jobs and absorb these CRWs post-training, and ensure continued professional development. This absence of capacity is particularly concerning because there is currently poor career pathing and continuing professional development for community-based rehabilitation workers (10, 11). Consistent with the WHO Rehabilitation 2030, CRWs' training programs in the rest of the African continent are critical avenues for scaling up and building capacity of human resources to meet rehabilitation demands (13). A responsive strategy here could be to mobilize African governments to open up posts for this workforce not only in Ministries of Health but across the sectors playing a role in community-based inclusive development implementation. Integral to this planning strategy, is the need for intersectoral budgeting particularly if we are to effectively implement disability inclusive development as a broad strategy (40).

The second point of discussion relates to the slowly changing mode of rehabilitation training. While programmes are starting to follow blended online modes of learning, we did not find programs transitioning toward community-based training on rural sites. This rural training in the community, has worked for medical student training. Increasingly, medicine as well as the allied health professions have been training more students at (peri-urban, urban and rural) sites away from the tertiary academic health center (42). Some studies in South Africa have identified benefits for such a decentralized clinical training for students, the health services and the community. For instance, Van Schalkwyk et al. (44) have argued that rural training of health professionals enhances rural practice preparedness. Additionally, it has a strong workforce imperative as it enhances the likelihood of such student working in these rural contexts (42, 44). This decentralized training happening at the community level could be a possible strategy to explore for training community-based rehabilitation workers through accreditation of higher education institutions. However, this strategy requires consistent commitment of financial and human resources for the training at community level, more trainers and facilities. It would mean that some hospitals or clinics as well as Non-Governmental Organizations (NGOs)/ Disabled People's Organizations (DPOs) become accredited sites for such training in order to build more capacity and professional learning of rehabilitation practitioners to serve as supervisors.

The capacity building of transdisciplinary supervisors requires that there is availability of relevant practitioners in the training communities. It has been common practice for CRWs to be supervised by managers with little or no experience and knowledge of rehabilitation (45). De Villiers et al. (42) found that availability of human resources who are willing, committed and motivated to train in decentralized training plays an integral role. But, for rehabilitation practice in rural contexts, the limited availability of workforce as well as limited knowledge of CBR (11) remain ongoing challenges which influence poor supervision issues and insufficient support of this cadre (12, 46). Gamiet and Rowe (43) argue that, with positive perceptions toward rehab care workers by rehabilitation practitioners related to strengthening CBR and PHC and sufficient training of supervisors, these CRWs could receive appropriate support. It may be that we need to also invest in recruiting and training supervision human resource through train-the-trainer programs as part of continued professional development. Likewise, the post-graduate programs aimed at strengthening skills of rehabilitation practitioners need to foreground rural practice.

The third point of the situational mapping overview results relates to the extent that training programs address competencies required for context specific rural settings with specific reference to the complexities that rural contexts pose to the practice of CRWs. Competency frameworks are an increasingly popular development tool to develop clinical governance, performance management, and professional development in health care. However, to date there is a dearth of information relating to specialist competencies for rehabilitation workers in remote and rural environments (1). While most emphasis on competencies seems to be placed on facilitating functioning, providing basic rehabilitation care and follow-up, less emphasis seems to be placed om understanding community-based rehabilitation as a community inclusive development strategy and facilitating community participation, advocacy and capacity building in the learning outcomes listed by training programs. Competencies for CBR workers in less resourced settings include disability knowledge, basic clinical skills, communication skills, management skills, and cultural competence (14, 35, 36). Being able to facilitate collaborative relationships is an essential skill required by mid-level rehabilitation workers in order for them to build and maintain partnerships necessary for program sustainability (39, 47, 48). These particular competencies are fairly represented in all the training programs included in this overview. Competencies focusing on the implementation of rural and remote service delivery, understanding disability and diversity (25, 30, 49) were however under-represented in programme outcomes.

Booyens et al. (25) argue that the interventions of CRWs need to document, target, measure and monitor the many barriers to inclusion and participation as a result of the complexity of impairments, social issues and power imbalances in rural contexts. However, these authors also ask whether these workers in rural communities necessarily require specialist training, or whether generic workers could be adequately trained and equipped for the complexities of rural contexts (25). This is an important question given that, as literature shows, people with disabilities in rural areas are subjected to institutional and environmental exclusion (25, 50, 51). Therefore, acquiring specific skills which capacitate CRWs to enable empowerment, restore dignity and humanity of people with disabilities as well as facilitate eradication of social, economic and institutional barriers embedded in rurality may require both general and targeted training.

The former is already addressed by the programmes found in this study. We therefore argue that targeted training ought to specifically speak to the links between disability, poverty, rurality, human rights and inclusion. We suggest that, one way in which the needed targeted training for CRWs in rural communities could address this link is to explicitly facilitate critical reflexivity around the complexities of rurality and disability. This is a significant shortcoming noted in the current learning outcomes and competencies for the various programmes listed in this review. Engaging in critical reflexivity on these complexities may aid CRWs to interrogate their own values and identify nuanced strategies needed to work toward social inclusion (52). Likewise, the training of rehabilitation practitioners/professionals who will be responsible for supervision of CRWs needs to equip these practitioners with critical reflexivity skills, particularly within a rural context, if they are to contribute to developing their practice.

## Conclusion

Based on this situational mapping overview, there is a need for an articulated process to develop community-based training of CRWs who will address the unmet rehabilitation needs in rural contexts of Southern Africa. This education and training of CRWs needs to be standardized while allowing flexibility for relevance, core specialist competencies for rural contexts and assessment standards to be developed. These rural practice specialist competencies of CRWs must include critical reflexivity, intersectoral collaborative practice and advocacy skills to enable these workers to address the prevailing attitudinal, systemic, environmental and institutional barriers facing people with disabilities and their families in rural settings. Moving to community orientated rural training may also require a different set of competencies for supervisors of these workers, where they may need to manage a team of CRWs who are active in the community. The competencies must include interprofessional practice, an improved understanding of community based rehabilitation as a community based inclusive development strategy as well as advocacy skills to be able to mobilize for resources of these workers at the community level.

As the supervision and mentoring relationship between CRWs and professionals is an important one, it would be necessary for institutions that offer respective training for this cadre of workers i.e., TVET colleges, Universities of Technology and universities, to explore how they could collaborate in fostering partnerships in joint training. This implies that inter-institutional as well as trans-disciplinary training could be facilitated as a key strategy for more training. Through such partnerships, opportunities for fieldwork placement and service-learning experiences as well as professional development to facilitate upskilling of CRWs could also be explored and formalized. These forms of coordinated commitments and actions are significant considerations toward developing training and maintaining a sustainable CBR workforce. This is particularly important for expanding and decentralizing the institutionalized services of rehabilitation to the community level.

Articulation is one strategy of starting this decentralized training. For instance, the first and second years of professional training could include CRWs and be based in the community. This would mean both the professionals and CRWs get an opportunity to develop competencies together and learn collaborative practice. Drawing from the issues that CRWs often have to address, the training has to emphasize on advocacy skills, rural practice and inclusive development skills.

The identified limitations of this overview include the acknowledgment that there may be other CRWs' training programmes available which are either not packaged as formal or not accredited or may be formalized but not accessible via the internet. These programmes may have been left out of this overview but may be helpful to inform the strengthening of accessible rural rehabilitation practice.

## Data Availability Statement

The original contributions presented in the study are included in the article/supplementary materials, further inquiries can be directed to the corresponding author.

## Ethics Statement

Ethics approval was obtained from the Stellenbosch University Health Research Ethics Committee (HREC Reference No: X19/03/007). The Stellenbosch University Health Research Ethics Committee provided a waiver for written informed consent for this study, as the study was a desktop review and did not involve dealing with any human subjects.

## Author Contributions

LN and LH-A led the conceptualization and writing of the various drafts of the manuscript. RT, LN, and LH-A conducted the search. All authors assisted with editorial and conceptualization of the paper. All authors contributed to the article and approved the submitted version.

## Conflict of Interest

The authors declare that the research was conducted in the absence of any commercial or financial relationships that could be construed as a potential conflict of interest.
